# Action of Saline Purgatives

**Published:** 1887-07

**Authors:** 


### Action of Saline Purgatives.

M. G. Leubuscher offers the following conclusion, in regard to the action of saline purgatives, after a series of experimental researches in this line :

" 1. That an exaggeration of the peristaltic movement of the intestine only plays a secondary part in the action of saline purgatives.

"2. In whatever matter saline purgatives may be introduced into the intestine, the intestine becomes the site of a great secre. tion of liquid, which is the principal cause of the purgative action.

" 3. It is impossible to claim for saline purgatives that they act as a barrier to re-absorption.

" 4. Saline purgatives introduced into the circulation in sufficient quanty produce constipation."--*Edinburgh Medical Journal.*

